# Etiological Profile and Antibiotic Susceptibility of Urinary Isolates Causing Urinary Tract Infections in Patients Attending a Tertiary Care Hospital in Rajkot, Gujarat

**DOI:** 10.7759/cureus.81633

**Published:** 2025-04-02

**Authors:** Mayuri Bhise, Kinjal Chauhan, Garima Anandani, Ashwini Agarwal

**Affiliations:** 1 Microbiology, All India Institute of Medical Sciences, Rajkot, Rajkot, IND; 2 Pathology, All India Institute of Medical Sciences, Rajkot, Rajkot, IND

**Keywords:** antibiogram, antimicrobial resistance, antimicrobial susceptibility, urinary tract infections, uropathogens

## Abstract

Objectives: This study aims to determine the occurrence of urinary tract infections (UTIs) and the bacteriological spectrum in urine samples, as well as to assess the antibiotic susceptibility patterns of the isolates.

Materials and methods: A retrospective study (October 2022 to October 2024) was conducted in the microbiology department, analyzing urine sample records using biochemical techniques and antimicrobial susceptibility testing guided by the Clinical and Laboratory Standards Institute. Data analysis was performed using SPSS Statistics version 23 (IBM Corp., 2015).

Results: Among 1,027 samples, 13.43% yielded positive cultures. Most infections occurred in females (64.49%), particularly those aged 51-70 (56%). Gram-negative bacteria (89.13%), predominantly *Escherichia coli* and *Klebsiella pneumoniae*, were more common than Gram-positive bacteria (9.42%). Effective antibiotics included fosfomycin (85.97%), gentamicin (90%), and nitrofurantoin (77.6%).

Conclusions: UTIs in this region are largely caused by Gram-negative bacteria, primarily *Escherichia coli*. Nitrofurantoin and fosfomycin are recommended for initial treatment. These findings support the development of empirical treatment protocols for the region.

## Introduction

Urinary tract infections (UTIs) rank among the most common and troublesome conditions in the realm of infectious diseases, impacting both outpatient and hospitalized individuals [1]. These infections pose a major healthcare challenge by increasing illness rates, driving up medical expenses, extending hospital stays, and diminishing the quality of life for those affected [2]. Acknowledging the seriousness of this issue, healthcare professionals and policymakers are increasingly concentrating on devising strategies and implementing preventive measures to effectively tackle UTIs. *Escherichia coli* is the bacterium most frequently found in UTIs, followed by *Klebsiella pneumoniae*, *Staphylococcus species*, *Proteus* species, *Pseudomonas aeruginosa*, *Enterococcus species*, and *Enterobacter* species, with the order of prevalence among these bacteria varying [3]. Worldwide, UTIs are responsible for 150 million cases each year, incurring healthcare costs of approximately $6 billion.

In rural and small-town areas, UTIs are often treated empirically due to limited resources for urine culture analysis, which can lead to inappropriate antibiotic use. Utilizing susceptibility data from nearby microbiological facilities is crucial for guiding the empirical selection of antibiotics. Rising drug resistance among uropathogens necessitates regular antibiotic susceptibility testing [4,5].

The antibiotic sensitivity of bacterial uropathogens varies over time and across different regions. Therefore, susceptibility screening in every area is essential to generate current and comprehensive epidemiological data [6]. Unfortunately, there is a lack of comprehensive exploration into the resistance profile of community-acquired uropathogens in different geographical areas of India [7]. In regions without microbiological facilities or where they are prohibitively expensive, UTIs are treated empirically.

This study aims to address the knowledge gap regarding the bacterial spectrum and antimicrobial susceptibility patterns of uropathogens in Rajkot, Gujarat, India. The knowledge gained from this study is essential for formulating an antibiotic policy for our institute [8].

## Materials and methods

Study setting

This retrospective study was carried out in the Department of Microbiology at a tertiary care hospital in Rajkot, located in the western region of India. The study analyzed data collected over two years from October 2022 to October 2024. The dataset was meticulously compiled from the microbiology laboratory's records, which serve as a paper-based repository of laboratory results, ensuring that comprehensive and accurate records were utilized.

Inclusion and exclusion criteria

UTI cases with only significant colony count (≥105 CFU/ml) of all the age groups and both sexes visiting the tertiary care hospital. Urine cultures showing no growth, mixed bacterial growth, or insignificant growth were excluded from the study.

Study population

The study was a retrospective review of the urine cultures done in the department of microbiology of the tertiary care hospital for two years from October 2022 to October 2024. Ethical approval for the study was secured from the Institutional Ethics Committee of All India Institute of Medical Sciences, Rajkot (approval number: AIIMS/RAJKOT/IEC/5th/ER/13), underscoring adherence to ethical standards and ensuring the protection of patient rights.

The study included all urine samples received during the defined period from all types of patients. It involved only a review of the sample processing registers for that period, analyzing data of 1027 urine samples. From the register, inpatient and outpatient samples were identified. Though it was very difficult to completely ascertain that all inpatient samples are hospital-acquired UTIs, for the estimation of UTIs acquired in the community and hospital, all the outpatient samples have been treated as community-acquired UTIs. In addition, since the register doesn’t have the details of the clinical syndrome, we assume that all the outpatient samples are from simple/uncomplicated symptomatic cystitis, as without the symptoms, patients are not sent for urine culture.

Sample collection and processing

Bacterial isolates with significant colony count (≥105 CFU/ml) were identified using standard microbiological methods, examining both cultural and biochemical characteristics. A series of biochemical tests, including indole, citrate utilization, urea hydrolysis, and carbohydrate fermentation, among others, were performed to confirm the identity of the isolates [9-11].

The Kirby-Bauer disc diffusion method was employed to evaluate the antibiotic susceptibility of the bacterial isolates. E-test was used for vancomycin susceptibility testing. Antibiotic discs were selected based on therapeutic relevance, and interpretations of susceptibility were carried out per Clinical and Laboratory Standards Institute guidelines [12,13].

Statistical analysis

Data from culture results and antibiotic sensitivity patterns were meticulously entered into a Microsoft Excel sheet (Microsoft Corp., Redmond, WA, USA) to ensure accuracy and organization. SPSS Statistics version 23 (IBM Corp. Released 2015. IBM SPSS Statistics for Windows, Version 23.0. Armonk, NY: IBM Corp.) was employed for data analysis. Descriptive statistics, including frequency, proportions, and percentages, were calculated to analyze and present the data.

## Results

**Figure 1:**
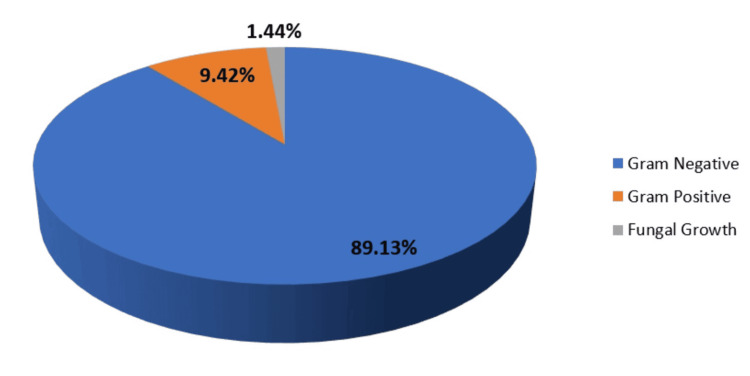
Culture positivity rates in cases of UTIs UTIs: urinary tract infections

Of a total of 1027 urine samples received and processed, 138 organisms were isolated from 1027 samples, yielding positive culture rates of 13.43% (Figure 1).

Out of 138 organisms, a very low isolation rate of Candida species (n=2, 1.44%) was observed in this study. The rest of the samples included 600 (58.42%) reported as no growth, 18 (1.75%) as contaminants, 57 (5.55%) as polymicrobial flora, and 214 (20.83%) as insignificant growth. Of the total 1027 urine samples received, 866 (84.32%) were from outpatient department (OPD) patients, and 161 (15.67%) were from inpatient department (IPD) patients. Among the 136 culture-positive patients, 91 (66.91%) were OPD patients, and 45 (33.09%) were IPD patients (Table 1).

**Table 1:** OPD and IPD distribution of culture-positive isolates OPD: outpatient department, IPD: inpatient department

	OPD		IPD	Total
Escherichia coli				
Male	17 (56.66%)		13 (43.33%)	30
Female	40 (74.07%)		14 (25.92%)	54
Klebsiella pneumoniae				
Male	6 (100%)		0	6
Female	16 (72.72%)		6 (27.27%)	22
Pseudomonas aeruginosa				
Male	1 (25%)		3(75%)	4
Female	0		0	0
Staphylococcus aureus				
Male	2 (40%)		3 (60%)	5
Female	2 (66.66%)		1 (33.33%)	3
Enterococcus species				
Male	1 (100%)		0	1
Female	1 (25%)		3 (75%)	4
Others				
Male	1 (50%)		1 (50%)	2
Female	4 (80%)		1 (20%)	5

**Figure 2:**
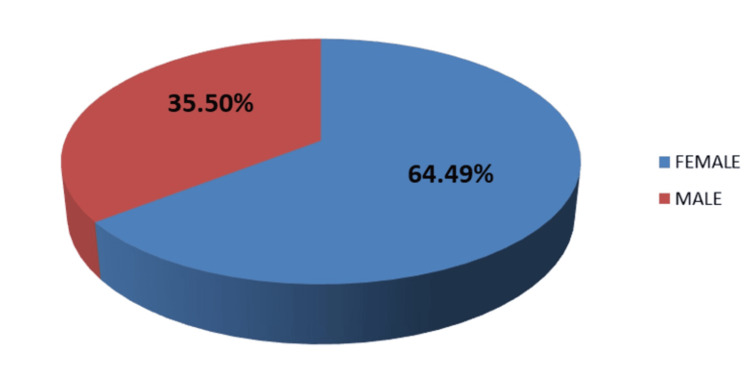
Distribution of culture-positive patients according to gender

The frequency of UTIs is higher in females (n=89, 64.49%) than in males (n=49, 35.50%) (Figure 2).

**Figure 3:**
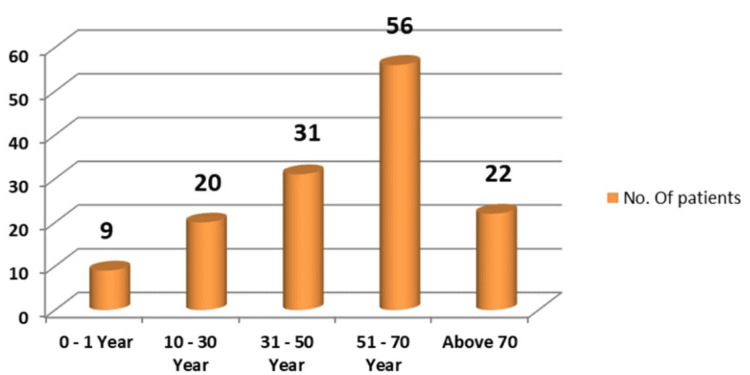
Distribution of patients according to their age group

In our study, the age ranged between one year and 90 years. The most common age group was 51-70 years (n=56, 40.57%), followed by 31-50 years (n=31, 22.46%). The mean age was 53.60 ± 17.55 years (Figure 3).

**Figure 4:**
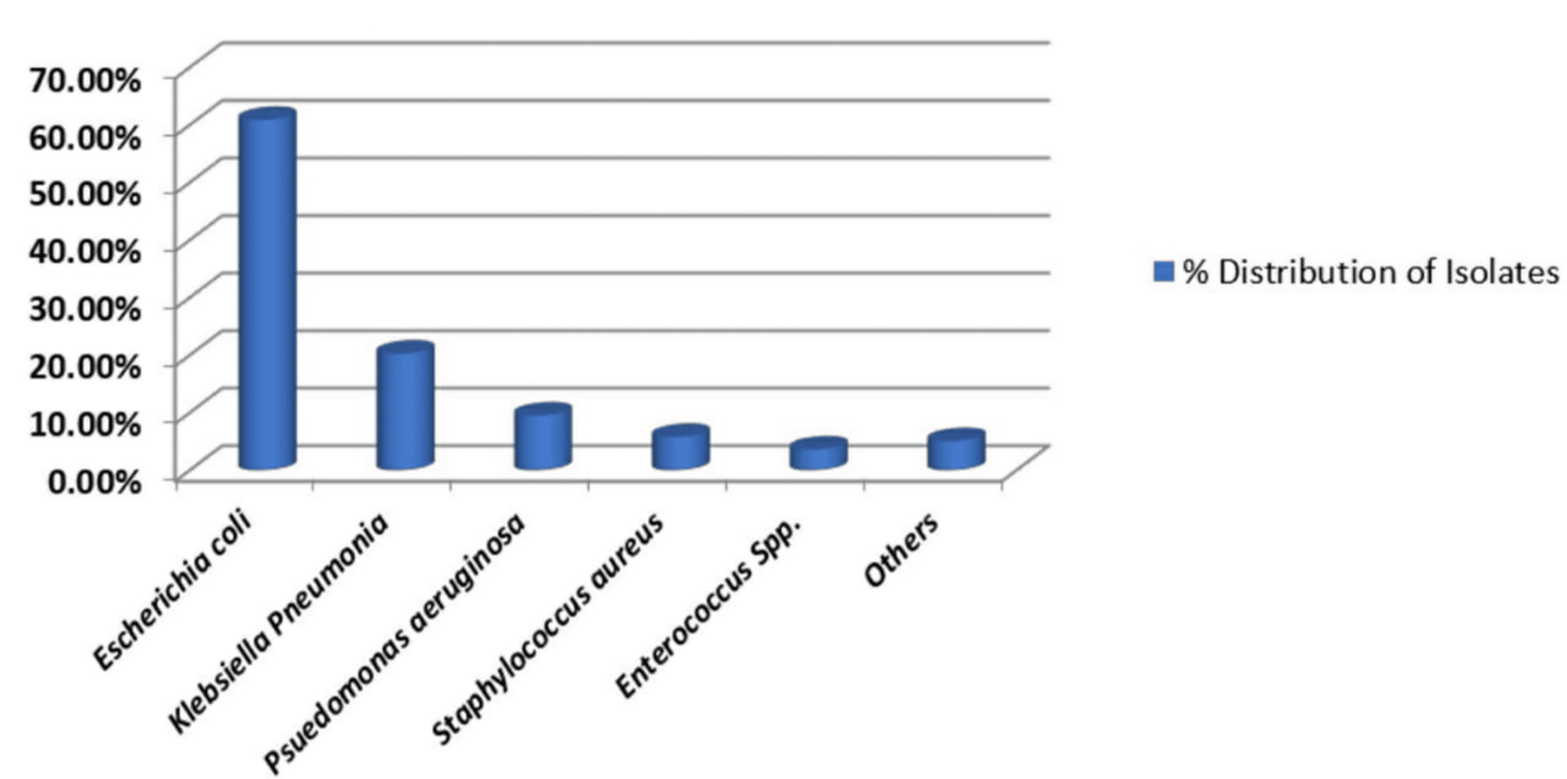
Percentage distribution of culture-positive isolates

Among the UTI-causing agents, Gram-negative organisms (n=123, 89.13%) were more predominant than Gram-positive organisms (n=13, 9.42%). Among Gram-negative isolates, *Escherichia coli* was the most common (n=84, 60.86%), followed by *Klebsiella pneumoniae* (n=28, 20.28%), *Pseudomonas aeruginosa* (n=4, 9.52%), and others (n=7, 5.072%) (Figure 4).

**Figure 5:**
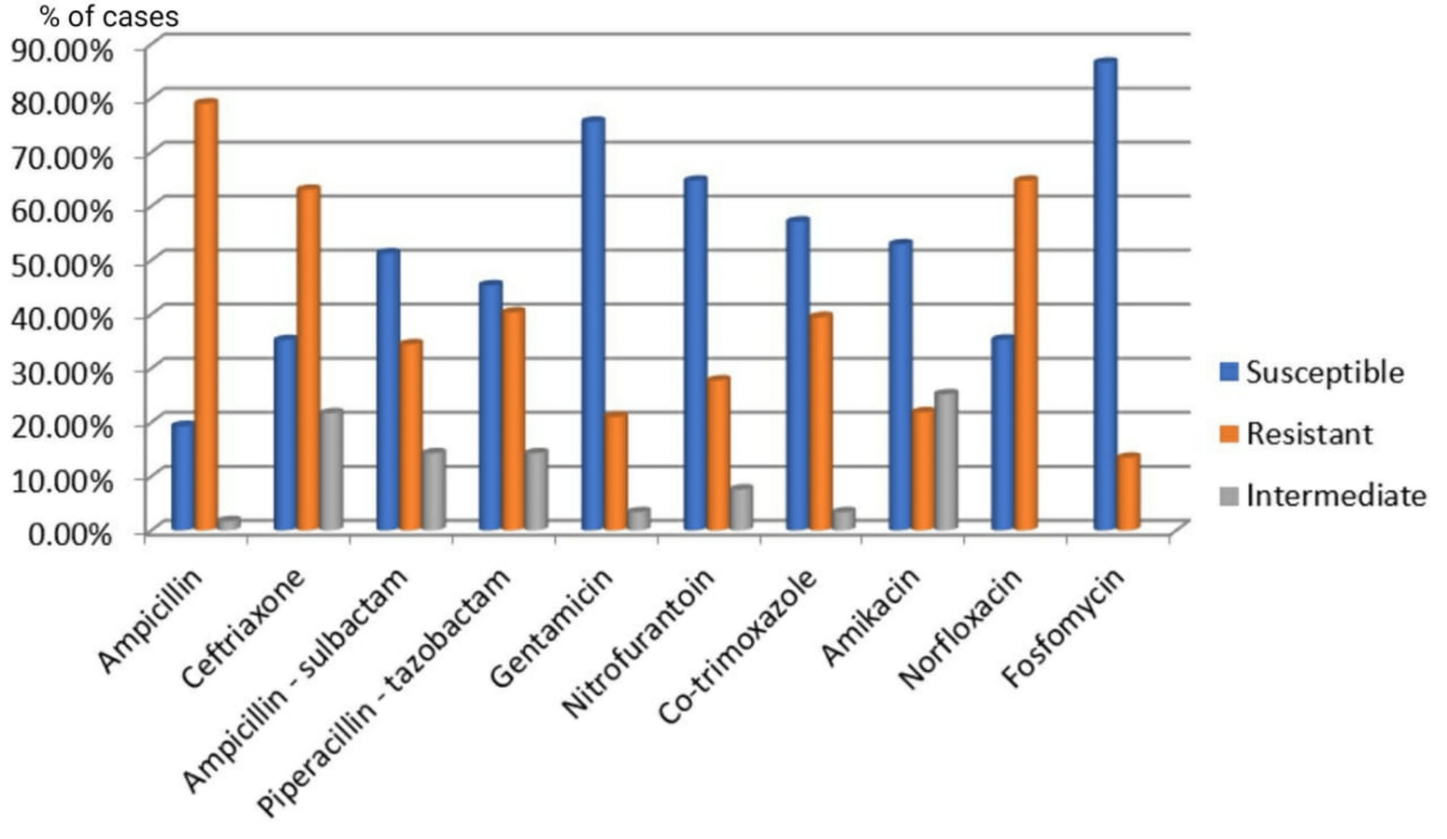
Antibiotic sensitivity pattern of Gram-negative isolates (Enterobacteriaceae)

**Figure 6:**
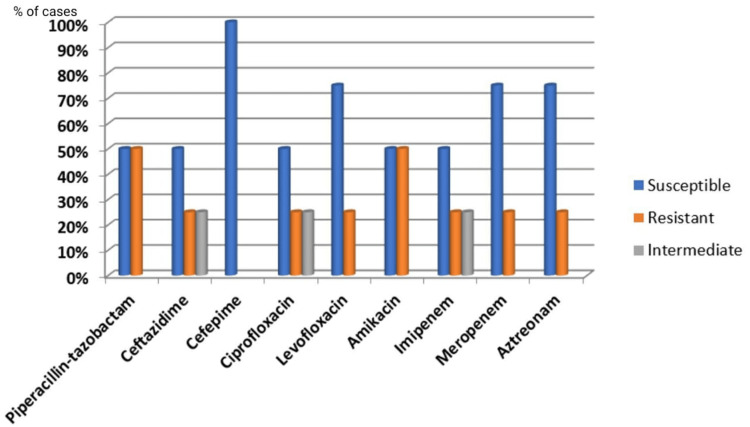
Antibiotic sensitivity pattern of Gram-negative bacilli (non-Enterobacteriaceae)

The most prevalent urinary tract pathogens in both genders in our study were *Escherichia coli* and *Klebsiella pneumoniae*. Among Gram-positive organisms, *Enterococcus* species (n=4, 57.14%) were more predominant in females than *Staphylococcus aureus* (n=3, 42.85%), while in males *Staphylococcus aureus* (n=5, 83.33%) was more predominant than *Enterococcus* species (n=1, 16.66%). The Gram-negative isolates were resistant to the primary therapeutic agents, ampicillin, ceftriaxone, norfloxacin, and cotrimoxazole, which are commonly employed for treating UTIs, as per findings from the antibiotic sensitivity tests. Fosfomycin (n=103, 86.55%), gentamicin (n=90, 75.63%), and nitrofurantoin (n=77, 64.70%) were highly sensitive in treating *Escherichia coli* isolates. Furthermore, other Gram-negative isolates exhibited 100% sensitivity to cefepime and moderate sensitivity to carbapenems (~75%), ceftazidime (n=2, 50%), and piperacillin-tazobactam (n=2, ~50%) (Figures 5-6).

**Figure 7:**
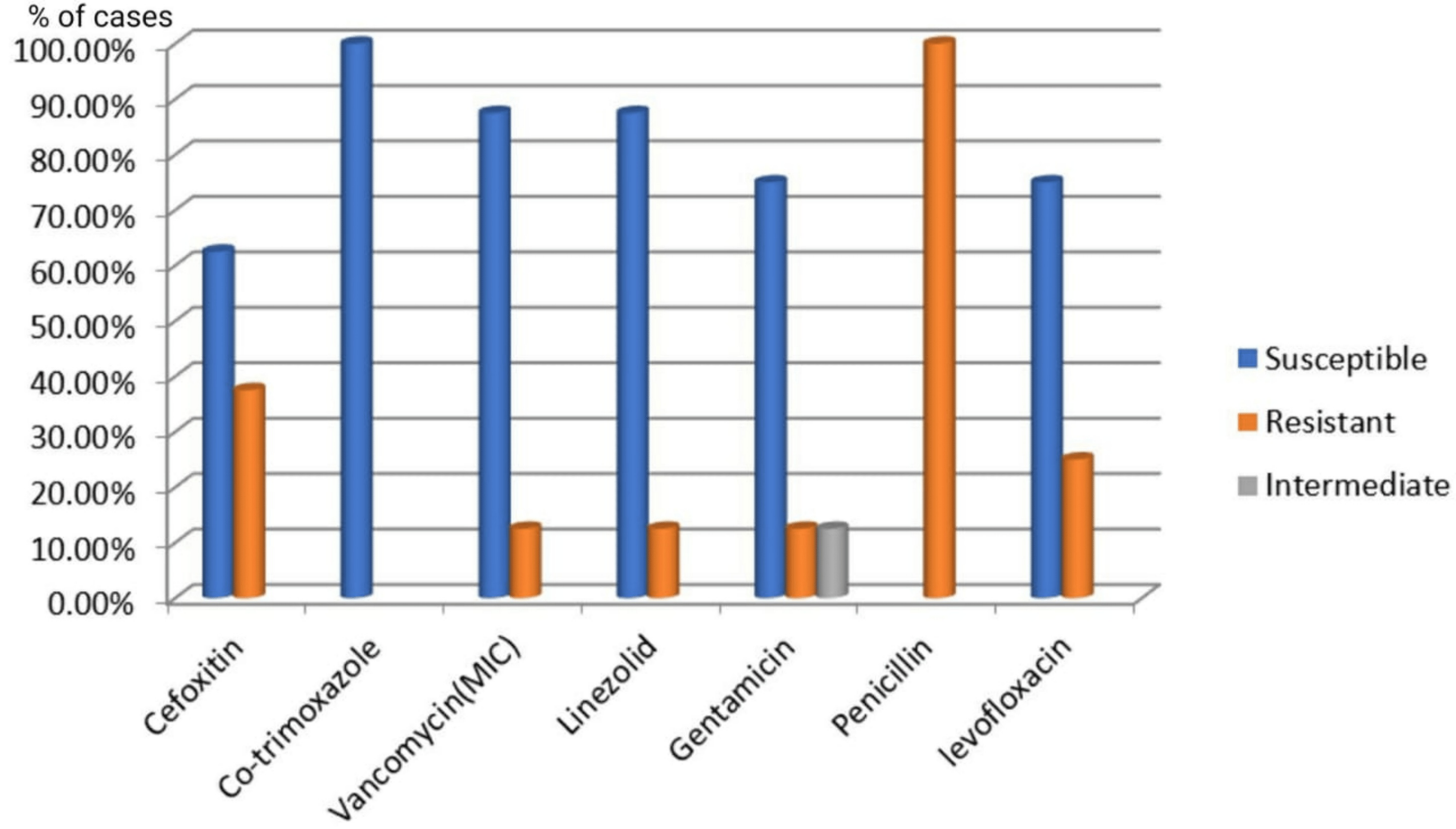
Antibiotic sensitivity pattern of Gram-positive cocci (Staphylococcus aureus)

**Figure 8:**
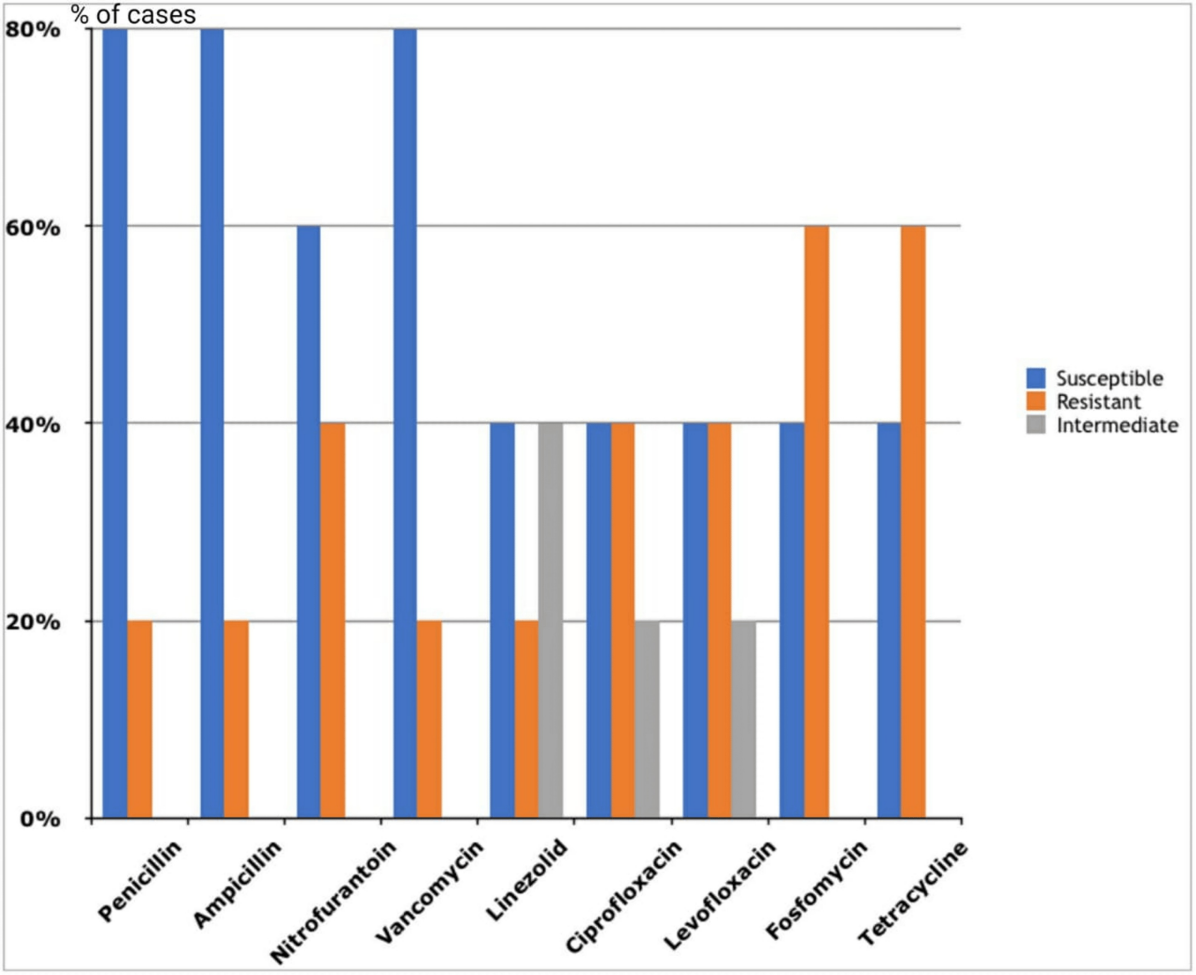
Antibiotic sensitivity pattern of Gram-positive cocci (Enterococcus spp.)

Among the Gram-positive organisms, all *Staphylococcus aureus* isolates were sensitive to cotrimoxazole (n=8, 100%), linezolid (n=7, 87.5%), and vancomycin (n=7, 87.5%). Of the eight *Staphylococcus aureus* isolates, three (37.50%) were methicillin-resistant *Staphylococcus aureus*, while five (62.50%) were methicillin-sensitive *Staphylococcus aureus*. *Enterococcus* species isolates were sensitive to vancomycin (n=4, 80%) and showed moderate sensitivity to nitrofurantoin (n=3, 60%) (Figures 7-8).

## Discussion

Bacterial infections of the urinary tract are a frequent reason for individuals to seek medical care in the community [14,15]. The causes, mechanisms, and antibiotic resistance patterns of uropathogens have changed over time and vary by location, and this trend is expected to continue. Identifying the organism and determining its antibiotic susceptibility are essential for treating UTIs. Successful treatment of bacterial UTIs often depends on pinpointing the type of organism responsible for the infection and choosing an effective antibiotic for that specific organism [16]. This highlights the necessity of strong collaboration between clinicians and microbiologists. In the current study, 138 out of 1027 urine samples from patients suspected of having UTIs (13.43%) showed significant pathogens. Our results align with Indian studies from Aligarh [17] and Jaipur [18], which reported lower culture positivity rates of 10.86% and 17.19%, respectively. Consistent with other research, our study found a higher incidence of UTIs in females (n=89, 64.49%) compared to males (n=49, 35.50%) [19,20]. Factors such as the proximity of the urethral opening to the anus, a shorter urethra, sexual activity, incontinence, and improper toilet habits may contribute to the higher UTI rates in females. In elderly males, the increased incidence of UTIs may be linked to a higher prevalence of benign prostate enlargement and neurogenic bladder [21]. Other studies support these findings, suggesting that prostate disease in older males contributes to the higher UTI rates.

The frequency of UTIs rises with advancing age [17]. In this study, participants' ages ranged from one to 90 years, with the majority (n=56, 40.57%) falling within the 51-70 age bracket, and the average age being 53.60 ± 17.55 years. These results align with the findings of Raval et al. [22] from Vadodara, India. Among the pathogens responsible for UTIs, Gram-negative bacteria (n=123, 89.13%) were more prevalent than Gram-positive bacteria (n=13, 9.42%). The higher occurrence of Gram-negative bacteria from the *Enterobacteriaceae* family in UTIs can be linked to factors such as their ability to adhere to the uroepithelium through urogenital mucosa colonization using adhesins, pili, fimbriae, and P-1 blood group phenotypic receptors [23]. In both genders, *Escherichia coli* and *Klebsiella pneumoniae* were the most common urinary tract pathogens, consistent with studies by Kumar et al. and Smita et al. [24,25]. Among Gram-positive bacteria, *Enterococcus* species (n=4, 57.14%) were more common in females than *Staphylococcus aureus* (n=3, 42.85%), whereas in males, *Staphylococcus aureus* (n=5, 83.33%) was more prevalent than *Enterococcus* species (n=1, 16.66%). All *Staphylococcus aureus* isolates were sensitive to cotrimoxazole (n=8, 100%), linezolid (n=7, 87.5%), and vancomycin (n=7, 87.5%). *Enterococcus* species showed complete sensitivity to vancomycin (n=4, 80%) and moderate sensitivity to nitrofurantoin (n=3, 60%). Antibiotics demonstrated high sensitivity rates to Gram-positive bacteria in our study, consistent with another research [18,26].

When managing UTIs, selecting appropriate antibiotics is essential for optimal outcomes. Gram-negative isolates showed significant resistance to commonly used UTI treatments such as ampicillin, ceftriaxone, norfloxacin, and cotrimoxazole, according to antibiotic sensitivity tests. Fosfomycin (n=103, 86.55%), gentamicin (n=90, 75.63%), and nitrofurantoin (n=77, 64.70%) were highly effective against *Escherichia coli* isolates. Additionally, other Gram-negative isolates were completely sensitive to cefepime and moderately sensitive to carbapenems (~75%), ceftazidime (n=2, 50%), and piperacillin-tazobactam (n=2, 50%). These findings suggest that fosfomycin, gentamicin, and nitrofurantoin are effective for treating *Escherichia coli* infections, while carbapenems, cefepime, and piperacillin-tazobactam may be suitable for other Gram-negative bacteria. Previous studies in India have shown that nitrofurantoin and fosfomycin are effective oral initial treatments for community-acquired UTIs, with fosfomycin primarily used for *Escherichia coli* infections [18,27]. The low isolation rate of *Candida* species (1.44%) in this study may be influenced by factors such as urinary catheterization and stenting, diabetes, immunocompromised status, hospitalization, and the use of broad-spectrum antibiotics.

Antibiotic resistance in India is significantly higher than in countries like the UK, USA, Australia, and South Africa [28]. Research shows a reduced effectiveness of empiric antibiotics against uropathogens causing UTIs, including cotrimoxazole and norfloxacin, as well as broad-spectrum antibiotics. Several factors contribute to the widespread use of antibiotics, such as self-medication, non-adherence to treatment, financial limitations, lack of patient education, the sale of antibiotics without proper prescriptions, inadequate monitoring of susceptibility patterns, weak regulatory controls, and insufficient patient education by pharmacists. Furthermore, antibiotics are often given before culture samples are obtained, and poor prescribing practices by doctors worsen the situation. These elements collectively lead to inappropriate antibiotic use in India, accelerating resistance development [29,30]. The Infectious Diseases Society of America advises that empirical antibiotic treatment for UTIs should be guided by regional susceptibility data, drug availability, and patient history. In India, resistance to bacterial uropathogens poses a major public health challenge. The absence of adequate microbiological laboratories in many Indian cities and towns results in fewer microbiological evaluations and increased empirical antibiotic use. Typically, urine samples are sent for microbiological testing only after treatment failure or recurrent infection. Given the evolving sensitivity patterns of various antibiotics, understanding the antibiogram of common isolates in a specific area or hospital is crucial for effective empirical treatment. Our study offered valuable insights into the common isolates and their antibiotic sensitivity and resistance patterns, aiding in the selection of appropriate drugs and reducing the burden of emerging antibiotic resistance in our hospital.

This study has some limitations. Firstly, the data for UTI-causing anaerobic bacteria, fungi, and viral agents is unavailable. The data for the phenotypic screening for extended-spectrum beta-lactamases, AmpC beta-lactamases, and metallo-beta-lactamases were also not available. There is also a lack of data on the confirmation of resistance by molecular methods.

## Conclusions

Our research has identified a considerable prevalence of Gram-negative bacterial isolates, with *Escherichia coli* identified as the primary pathogen responsible for UTIs, closely followed by *Klebsiella pneumoniae*. Additionally, we have performed an extensive evaluation of antibiogram data. In light of our findings, we recommend nitrofurantoin and fosfomycin as the first-line treatments for UTIs in our area. These recommendations will be applicable to both our inpatient and outpatient facilities, as well as other departments within our organization.

## References

[1] Medina M, Castillo-Pino E (2019). An introduction to the epidemiology and burden of urinary tract infections. Ther Adv Urol.

[2] Zeng Z, Zhan J, Zhang K, Chen H, Cheng S (2022). Global, regional, and national burden of urinary tract infections from 1990 to 2019: an analysis of the global burden of disease study 2019. World J Urol.

[3] Ahmed SS, Shariq A, Alsalloom AA, Babikir IH, Alhomoud BN (2019). Uropathogens and their antimicrobial resistance patterns: relationship with urinary tract infections. Int J Health Sci (Qassim).

[4] Flores-Mireles AL, Walker JN, Caparon M, Hultgren SJ (2015). Urinary tract infections: epidemiology, mechanisms of infection and treatment options. Nat Rev Microbiol.

[5] Al-Zahrani J, Al Dossari K, Gabr AH, Ahmed AF, Al Shahrani SA, Al-Ghamdi S (2019). Antimicrobial resistance patterns of uropathogens isolated from adult women with acute uncomplicated cystitis. BMC Microbiol.

[6] Daoud N, Hamdoun M, Hannachi H, Gharsallah C, Mallekh W, Bahri O (2020). Antimicrobial susceptibility patterns of Escherichia coli among Tunisian outpatients with community-acquired urinary tract infection (2012-2018). Curr Urol.

[7] Tryphena C, Sahni RD, John S, Jeyapaul S, George A, Helan J (2021). A retrospective study on the microbial spectrum and antibiogram of uropathogens in children in a secondary care hospital in Rural Vellore, South India. J Family Med Prim Care.

[8] Walia K, Ohri VC, Madhumathi J, Ramasubramanian V (2019). Policy document on antimicrobial stewardship practices in India. Indian J Med Res.

[9] Collee JG, Miles RS, Watt B (1996). Tests for the identification of bacteria. Mackie & McCartney practical medical microbiology, 14th edition.

[10] McCarter YS, Burd EM, Hall GS, Zervos M (2009). Laboratory diagnosis of urinary tract infections.

[11] Munoz-Dávila MJ, Roig M, Yagüe G, Blázquez A, Salvador C, Segovia M (2013). Comparative evaluation of Vitek 2 identification and susceptibility testing of urinary tract pathogens directly and isolated from chromogenic media. Eur J Clin Microbiol Infect Dis.

[12] Sleigh JD, Timbury MC (1986). Notes on medical bacteriology. Notes on Medical Bacteriology. Second ed. New York: Churchill Livingstone Inc., 1560 Broadway.

[13] Clinical and Laboratory Standards Institute (2025). Performance CLSI. Standards for Antimicrobial Susceptibility TestingWayne, PA: Clinical and Laboratory Standards Institute; 2024 CLSI supplement M100. M100—performance standards for antimicrobial susceptibility testing, 32nd edition.

[14] Kang CI, Kim J, Park DW (2018). Clinical practice guidelines for the antibiotic treatment of community-acquired urinary tract infections. Infect Chemother.

[15] Porter G, Grills N (2016). Medication misuse in India: a major public health issue in India. J Public Health (Oxf).

[16] Moue A, Aktaruzzaman SA, Ferdous N, Khalil RK, Das AK (2015). Prevalence of urinary tract infection in both outpatient department and in patient department at a medical college setting of Bangladesh. Int J Biosci.

[17] Akram M, Shahid M, Khan AU (2007). Etiology and antibiotic resistance patterns of community-acquired urinary tract infections in J N M C Hospital Aligarh, India. Ann Clin Microbiol Antimicrob.

[18] Sood S, Gupta R (2012). Antibiotic resistance pattern of community acquired uropathogens at a tertiary care hospital in jaipur, rajasthan. Indian J Community Med.

[19] Prakash D, Saxena RS (2013). Distribution and antimicrobial susceptibility pattern of bacterial pathogens causing urinary tract infection in urban community of meerut city, India. ISRN Microbiol.

[20] Odoki M, Almustapha Aliero A, Tibyangye J (2019). Prevalence of bacterial urinary tract infections and associated factors among patients attending hospitals in Bushenyi District, Uganda. Int J Microbiol.

[21] Malik S, Rana JS, Nehra K (2021). Prevalence and antibiotic susceptibility pattern of uropathogenic Escherichia coli strains in Sonipat region of Haryana in India. Biomed Biotechnol Res J.

[22] Raval R, Verma RJ, Kareliya H (2015). Clino‑pathological features of urinary tract infection in rural India. Adv Infect Dis.

[23] Terlizzi ME, Gribaudo G, Maffei ME (2017). Uropathogenic Escherichia coli (UPEC) infections: virulence factors, bladder responses, antibiotic, and non-antibiotic antimicrobial strategies. Front Microbiol.

[24] Kumar GL, Shreedevi S, Pratyusha Y, Balakrishna J, Lavanya V (2020). Bacterial profile and antibiogram of urine culture isolates in a teritiary care center. Int J Med Microbiol Trop Dis.

[25] Smita MS, Wavare SS, Gajul S, Sajjan AG (2019). Bacterial profile of urinary tract infections and antibiotic resistance pattern in a tertiary care hospital. Online J Health Sci.

[26] Kumar R, Dahiya SS, Hemwani K, Srivastava P (2014). Isolation of human pathogenic bacteria causing urinary tract infection and their antimicrobial susceptibility pattern in a tertiary care hospital, Jaipur, India. Int Res J Med Sci.

[27] Mohapatra S, Panigrahy R, Tak V (2022). Prevalence and resistance pattern of uropathogens from community settings of different regions: an experience from India. Access Microbiol.

[28] (2018). ResistanceMap. https://www.resistancemap.cddep.org/AntibioticResistance.php.

[29] World Health Organization (1996). World Health Organization. The World Health Report 1996. Geneva, Switzerland: World Health Organization. The world health report 1996: fighting disease, fostering development.

[30] World Health Organization (2017). World Health Organisation. National Action Plan on Antimicrobial Resistance (NAP‑AMR) 2017‑2021. New Delhi, India: World Health Organisation Country Office for India. India: national action plan on antimicrobial resistance (NAP-AMR) 2017-2021.

